# Circadian Modulation of the Cl^−^ Equilibrium Potential in the Rat Suprachiasmatic Nuclei

**DOI:** 10.1155/2014/424982

**Published:** 2014-05-18

**Authors:** Javier Alamilla, Azucena Perez-Burgos, Daniel Quinto, Raúl Aguilar-Roblero

**Affiliations:** ^1^División de Neurociencias, Instituto de Fisiología Celular, Universidad Nacional Autónoma de México, Apartado Postal 70-253, 04510 México, DF, Mexico; ^2^Department of Psychology, Neuroscience & Behaviour, McMaster University, Hamilton, ON, Canada L8S 4K1; ^3^McMaster Brain-Body Institute, St Joseph's Healthcare, Hamilton, ON, Canada L8N 4A6

## Abstract

The suprachiasmatic nuclei (SCN) constitute a circadian clock in mammals, where *γ*-amino-butyric acid (GABA) neurotransmission prevails and participates in different aspects of circadian regulation. Evidence suggests that GABA has an excitatory function in the SCN in addition to its typical inhibitory role. To examine this possibility further, we determined the equilibrium potential of GABAergic postsynaptic currents (*E*_GABA_) at different times of the day and in different regions of the SCN, using either perforated or whole cell patch clamp. Our results indicate that during the day most neurons in the dorsal SCN have an *E*_GABA_ close to −30 mV while in the ventral SCN they have an *E*_GABA_ close to −60 mV; this difference reverses during the night, in the dorsal SCN neurons have an *E*_GABA_ of −60 mV and in the ventral SCN they have an *E*_GABA_ of −30 mV. The depolarized equilibrium potential can be attributed to the activity of the Na(+)-K(+)-2Cl(−) (NKCC) cotransporter since the equilibrium potential becomes more negative following addition of the NKCC blocker bumetanide. Our results suggest an excitatory role for GABA in the SCN and further indicate both time (day versus night) and regional (dorsal versus ventral) modulation of *E*_GABA_ in the SCN.

## 1. Introduction

The main pacemakers implicated in the regulation of circadian rhythms in mammals are the suprachiasmatic nuclei (SCN) [1]. The SCN is divided into two distinct anatomical and functional subdivisions: the ventrolateral SCN or core, which receives most of the afferent inputs to the SCN from the retina, the median raphe nucleus, and the intergeniculate leaflet and has been implicated in entrainment to external cycles, and the dorsomedial SCN, or shell, which has been implicated in the pacemaker function and in the output of the circadian clock to the rest of the brain [2–4]. A molecular oscillator, involving translation-transcription loops of specific clock genes in SCN neurons, generates circadian overt rhythms from the pacemaker neurons as the spontaneous firing rate (SFR), metabolic activity, and synthesis and secretion of neurotransmitters and neuropeptides [5].

The most widespread neurotransmitter in the SCN is GABA, which colocalizes with different peptides in specific regions of the SCN. For example, vasopressin colocalizes with GABA in the dorsomedial region and vasoactive intestinal polypeptide colocalizes with GABA in the ventromedial region [6, 7]. The GABA synthesizing enzyme L-glutamic acid decarboxylase (GAD) and the concentration of GABA itself have distinct circadian rhythmicity in SCN neurons [8]. There is also circadian rhythmicity in the frequency of spontaneous inhibitory GABAergic postsynaptic currents [9, 10]. Furthermore, several studies indicate that GABA plays an important role in the entrainment of the circadian rhythms [11–13]; it also seems to couple the dorsal and ventral SCN in order to function as an integrated oscillator [14].

Wagner et al. [15] first described excitatory effects of GABA in SCN during daytime, which created some controversy about the role of GABA as a neurotransmitter in SCN neurons. Some studies indicate that GABA has its usual inhibitory functions [16–18], whereas others support excitatory actions of GABA in the SCN nuclei [19, 20]. Additionally, de Jeu and Pennartz [21] found excitatory effects of GABA during the night, Albus et al. [14] demonstrated excitatory actions of GABA in the dorsal part of the SCN during the day and night, Choi et al. [22] reported excitatory actions during the night in the dorsal SCN attributable to the Cl^−^ cotransporter NKCC1, and Irwin and Allen [23] found that GABA elicits opposite effects on SCN neuronal Ca^2+^ responses and that excitatory responses involve the NKCC1 cotransporter. In order to contribute to resolving this controversy, we characterized *E*_GABA_ in the two main topographical regions of the SCN (dorsal and ventral) at two different times of the day (around midday or midnight), using perforated and whole cell patch clamp recordings in coronal brain slices.

## 2. Materials and Methods

### 2.1. Animals and General Conditions

Male Wistar rats (35–45 days old, 100–120 g) were housed under 12 : 12 h light : dark cycle (lights on at 6:00, 400 lux; 22°+/−1°C) in a sound attenuated room for a week before the experiments. For experiments during the night, animals were maintained in a reversed 12 : 12 h light : dark cycle (lights on at 22:00 h, 400 lux) for 3 weeks before the experiments. Animals had food and water* ad libitum*. All the procedures were conducted according to the guidelines for use of experimental animals from the Universidad Nacional Autónoma de México in accordance with national laws (NOM-062-200-1999) and the guidelines from the Society for Neuroscience.

### 2.2. Slice Preparation

Rats were deeply anaesthetized with isoflurane 3 h after lights on (ZT 3) and their brains were quickly removed and placed in an ice cold low Ca^2+^ artificial cerebrospinal fluid (aCSF) containing (in mM): 126 NaCl, 2.5 KCl, 1.2 NaH_2_PO_4_, 4 MgCl_2_, 0.5 CaCl_2_, 26 NaHCO_3_, and 10 glucose, pH 7.38, 330 mOsm/L, oxygenated with 95% O_2_/5% CO_2_. For night recording, to avoid phase shifts induced by light, the brain extraction was done 1 h before lights off (ZT 11). Coronal slices (250–300 *μ*m) containing the SCN were cut on a vibratome (Vibratome, St. Louis, MO, USA) and transferred to a recovery chamber with fresh low Ca^2+^ aCSF at RT until use (at least 1 hour of recovery). The slices were then placed in the recording chamber and continuously perfused (2.5–5 mL/min) with oxygenated aCSF. The recording chamber solution was the same as the extraction solution except that CaCl_2_ was increased to 2.4 mM and MgCl_2_ reduced to 1.3 mM. SCN neurons were viewed and the recording electrodes were positioned by infrared Nomarski microscopy at 60x using a Nikon Eclipse 600 (Nikon, Melville, NY, USA) with a Dage MTI video camera and monitor.

### 2.3. Patch Clamp Recordings

Voltage clamp recordings were performed with either perforated or whole cell patch clamp techniques at RT (20–25°C). Recordings were made either between 5 and 10 h after lights on (ZT 5–10, day) or between 5 and 10 h after lights off (ZT 17–22, night). For perforated patch recordings, borosilicate electrodes (tip diameter, 1.0–1.5 *μ*m; 3–5 MΩ, WPI, Sarasota, FL, USA) were filled with intracellular solution containing (in mM): 143 K-gluconate, 2 KCl, 10 HEPES, and 0.5 EGTA, pH 7.38, adjusted with KOH, 275 mOsml/L. The antibiotic gramicidin was used in order to maintain the intracellular Cl^−^ concentration [Cl^−^]_i_ [24]. Gramicidin was dissolved in dimethyl sulfoxide (DMSO) and was prepared as a stock solution on the day of the experiment (10 mg/mL). The intracellular solution plus gramicidin (50 *μ*g/mL) was not filtered. The recording electrodes were backfilled with the gramicidin-containing solution and the tip of the electrode was filled with gramicidin-free solution. It usually took between 5 and 10 min to perforate the membrane and between 15 and 30 min to obtain a stable series resistance. Capacitance and series resistance were compensated by a minimum of 80%, and the seal was monitored throughout each experiment. Cells were discarded if input resistance was lower than 150 MΩ and/or access resistance exceeded 45 MΩ or changed more than 15%. Access resistance was usually ≤35 MΩ.

Two intracellular solutions were used for whole cell recordings. The first had a calculated *E*_Cl^−^_ of −30 mV (at 25°C) and contained (in mM): 72 KH_2_PO_4_, 36 KCl, 2 MgCl_2_, 10 HEPES, 1.1 EGTA, 0.2 Na_2_ATP, and 0.2 Na_2_GTP, pH 7.38 adjusted with KOH, 275 mOsmol/L. The other intracellular solution had a calculated *E*_Cl^−^_ of −60 mV (at 25°C) and contained (in mM): 111 KH_2_PO_4_, 12 KCl, 2 MgCl_2_, 10 HEPES, 1.1 EGTA, 0.2 Na_2_ATP, and 0.2 Na_2_GTP, pH 7.2, adjusted with KOH, 275 mOsmol/L. Pipettes had a tip diameter of 1.0–1.5 *μ*m and a resistance of 3–5 MΩ. Once a good seal was obtained (above 2 GΩ) between the recording electrode and the neuron, the membrane was disrupted by a gentle suction. Capacitance and series resistance were compensated and the seal was monitored similarly to perforated patch experiments. Neurons were discarded if input resistance was <150 MΩ and/ or if access resistances were >25 MΩ or changed more than 15% during the experiment. The average access resistance was less than 15 MΩ.

Recordings were made with an Axopatch 200B amplifier (Axon instruments, Foster City, CA, USA). Online data was collected with a custom made program in the LabView environment through a digital acquisition board (DAQ, National Instruments, Austin, TX, USA). Recordings were sampled at 10 kHz and filtered at 5 kHz.

### 2.4. Postsynaptic Currents

To examine spontaneous postsynaptic currents (sPSCs) at different membrane holding potentials (from −100 mV to +80 mV), QX-314 (5 mM) was added into the recording pipette solution in order to block Na^+^ currents. Pharmacological isolation of GABAergic sPSCs was accomplished by adding DL-2-amino-5-phosphonopentanoic acid (APV, 50 *μ*M) and 6,7-dinitroquinoxaline-2,3(1H,4H)-dione (DNQX, 10 *μ*M) into the recording aCSF. Bicuculline methiodide (10 *μ*M) was applied to abolish all the spontaneous synaptic inputs and to confirm that GABA produced the sPSCs. Bumetanide (10 *μ*M) was used to block NKCC1 in order to determine its contribution to *E*_GABA_. All drugs were purchased from Sigma (St. Louis, MO, USA).

### 2.5. Localization of Recorded Neurons in the Dorsal or Ventral Regions of the SCN

The neurons recorded were visualized using a 10x objective water immersion lens (Nikon) and marked in a representative drawing of the SCN (previously prepared) with the third ventricle at the center and the optic chiasm at the bottom, which resembles the coronal slice with the two SCN. When the experiment was finished, the schematic drawing was analyzed by an independent observer and neurons were categorized as ventral or dorsal according to Abrahamson and Moore [2].

### 2.6. Electrophysiological Data Analysis

Analysis of the synaptic currents was done with Mini Analysis 6.0.3 (Synaptosoft, Decatur, GA, USA). The *E*_GABA_ was estimated as follows: (1) for the average *E*_GABA_ obtained from the neurons at each group (SCN region/time of recording). The *E*_GABA_ in each neuron was calculated by the interception of zero current with its corresponding voltage value (mV). To calculate the zero current, we fitted a third order polynomial curve to the* I*-*V* plot using Origin 8 (Origin Lab, Northampton, MA, USA) and (2) by the interception of the zero current and its corresponding voltage (mV) obtained by the* I*-*V* curves shown in the figures. Each dot indicates the mean ± SEM current that was obtained from the neurons in the corresponding category (SCN region/time of recording). The line represents a third order polynomial curve fit to all data in the* I*-*V*. There were only minor discrepancies in the *E*_GABA_ data obtained by the two methods.

### 2.7. Statistical Analysis

The values are reported in text and tables as the range from the minimum to the maximal individual values, median, and mean ± SEM for each group. Normality tests (Shapiro-Wilk) were applied to the different samples (*E*_GABA_ sorted by the SCN region/time of recording) for perforated patch and whole cell recordings. The groups were not normally distributed (*P* < 0.05). Gaussian curves were fitted to the data graphed in frequency histograms. “Best fit” analyses suggested the presence of more than one population in the sample (*χ* ^2^,* R* ^2^). −50 mV was chosen to divide the samples for two reasons: (1) this value split the Gaussian curves (the tails of the curves overlapped around this point) and (2) the median for all the samples was equal or very close to −50 mV (Table 1).

Statistical analysis was done using GraphPad Prism 4.0 (GraphPad Sowftware, La Joya, CA, USA), Origin 8, and SPSS (IBM Corporation, Armonk, NY). Statistical tests were nonparametric (Wilcoxon test for matched pairs) unless otherwise stated. Kruskal-Wallis for independent samples was performed in those data that represent the predominant part of the sample (above or below −50 mV) for the experiments in perforated patch configuration. The *α* level was set at 0.05.

## 3. Results

**Figure 1:**
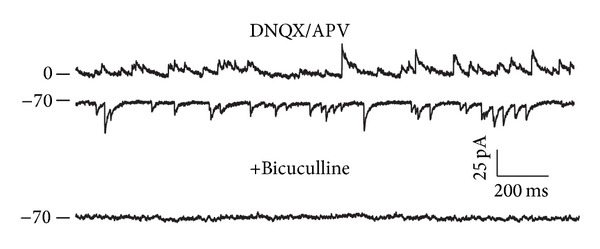
Spontaneous GABAergic postsynaptic currents in the suprachiasmatic nucleus. GABA sPSCs were pharmacologically isolated by administration of the ionotropic glutamate receptor antagonists, DNQX (10 *μ*M) and APV (50 *μ*M). Recordings at 0 mV and −70 mV are shown. Subsequent bicuculline administration (10 *μ*M) completely abolished the sPSCs at all membrane holding potentials. Example recordings obtained in a dorsal SCN neuron during the day period in perforated patch configuration.

A total of 178 SCN neurons were recorded; 97 neurons were recorded in perforated patch configuration and the remaining 81 in whole cell configuration. All experiments were carried out in the presence of DNQX and APV in order to isolate the GABAergic synaptic currents. At least 100 sPSCs were analyzed at each holding potential from −100 mV to +80 mV (20 mV steps). The GABAergic nature of the sPSCs was confirmed if they were completely abolished by bicuculline administration (Figure 1).

### 3.1. Perforated Patch Clamp Recordings

**Figure 2:**
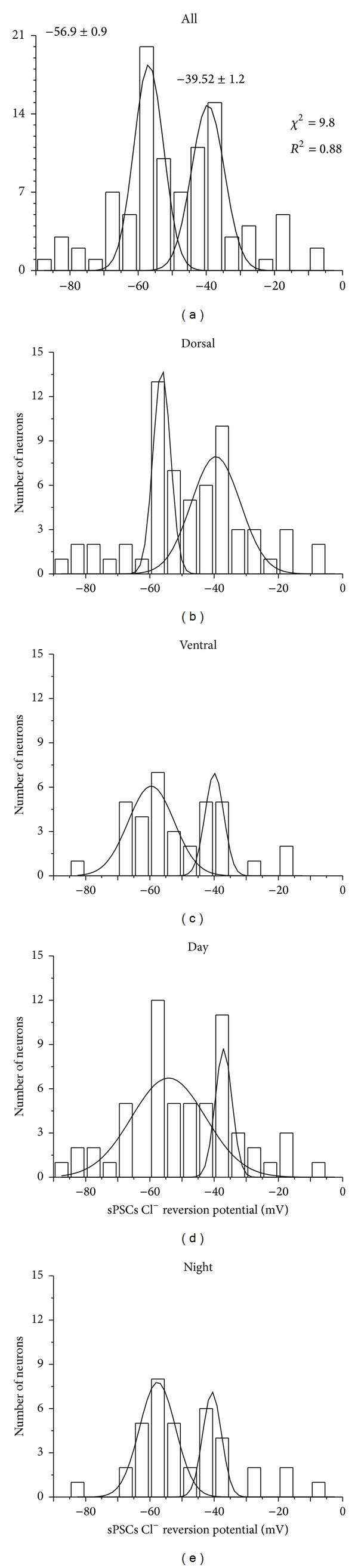
Frequency histograms of *E*_GABA_ obtained using perforated patch recordings. Gaussian fitting analyses revealed two populations in *E*_GABA_ distribution, belonging to (a) all the neurons recorded in different ZT and SCN regions, (b, c) *E*_GABA_ sorted by dorsal and ventral SCN regions, and (d, e) *E*_GABA_ grouped by ZT of recording.

**Figure 3:**
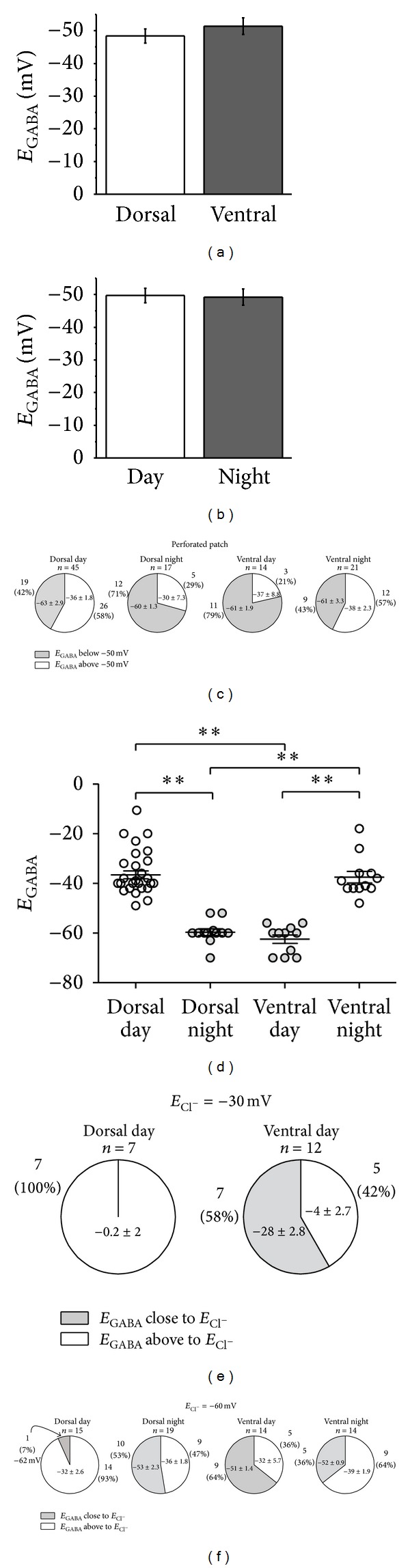
*E*_GABA_ distributions in the SCN. Comparison of all *E*_GABA_ values recorded in perforated patch configuration, grouped by SCN region (a) or ZT of recording (b). Values are shown in mean ± SEM. (c) Distribution of *E*_GABA_ separated by SCN region and ZT of recording. Experiments obtained in perforated patch configuration. (d) Statistically significant differences were found with Kruskal-Wallis test (*H* = 42.4, *P* < 0.0001). Analysis was performed with the prevailing part of the groups depicted in (c). Dunn's multiple comparison test indicated significant differences between the groups (*P* < 0.05). Distribution of *E*_GABA_ in neurons recorded in whole cell configuration with a theoretical *E*_Cl^−^_ of −30 mV (e) and *E*_Cl^−^_ of −60 mV (f).

The descriptive statistics of the equilibrium potential of sPSCs from all 97 neurons are shown in Table 1. No differences were found when the data was sorted by either SCN location (dorsal versus ventral, Figure 3(a)) or time of recording (subjective day versus subjective night, Figure 3(b)). Nevertheless, inspection of the frequency histogram of sPSCs equilibrium potential suggests the presence of more than one population, one above and one below −50 mV. This was examined further by fitting the frequency histogram to a two-peak Gaussian model; the results demonstrate a *χ* ^2^ = 9.8 and a* R* ^2^ = 0.88 (Figure 2(a)). Arrangement of the data by SCN location or time of recording showed in all cases a similar pattern of two partially overlapped Gaussian distributions around −50 mV as shown in Figures 2(b)–2(e).

**Figure 4:**
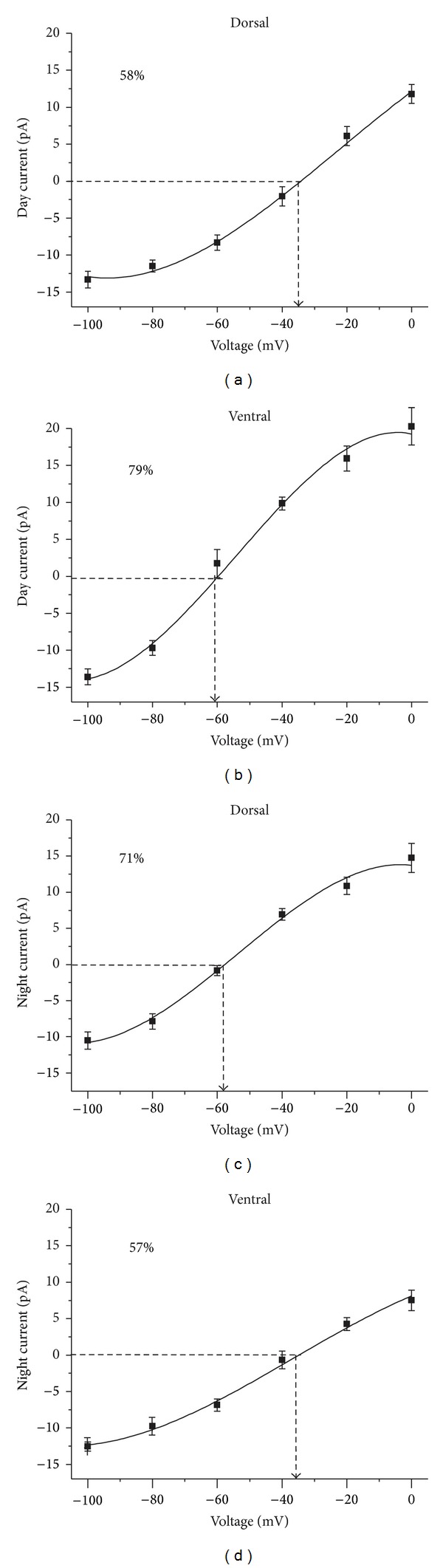
Diurnal and regional modulation of *E*_GABA_ from neurons recorded using perforated patch. During the day, the predominant equilibrium potential from the dorsal SCN neurons is −37 ± 2 mV (a) and in the ventral SCN is −61 ± 2 mV (b); during the night, the pattern reverses so that the predominant equilibrium potential from the dorsal SCN is −60 ± 1 mV (c) and in the ventral SCN is −38 ± 2 mV (d). Each panel shows the percentage of neurons contributing to each* I*-*V* curve.

**Figure 5:**
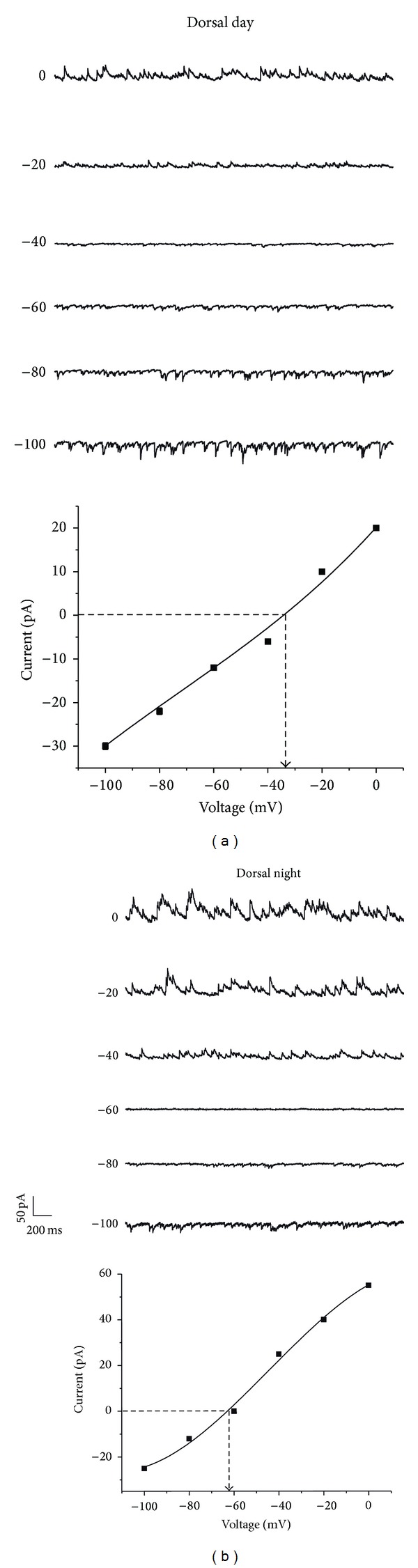
*E*_GABA_ in dorsal SCN region. (a) Perforated patch recording in a dorsal SCN neuron obtained during the day. *E*_GABA_ for this neuron was −33 mV, as shown in the* I*-*V* curve below. (b) Recording performed using perforated patch in a dorsal SCN neuron during the nocturnal ZT. The *E*_GABA_ for this example was −62 mV (*I*-*V* curve below).

**Figure 6:**
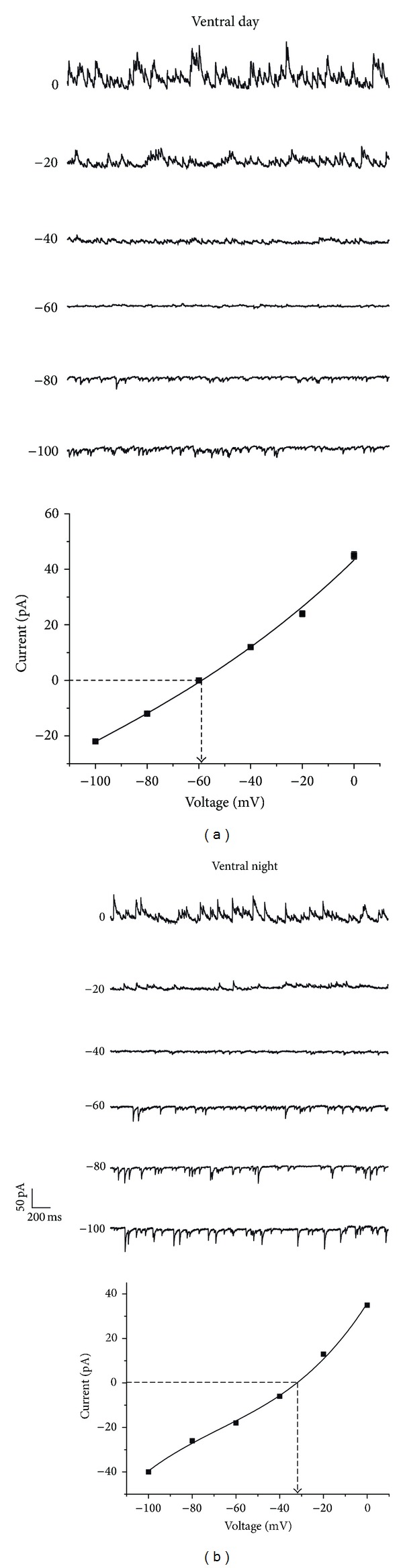
*E*_GABA_ in ventral SCN region. (a) Neuron recorded in perforated patch in a ventral SCN neuron during the day. *E*_GABA_ for this neuron was −58 mV (*I*-*V* curve below). (b) Perforated patch recording performed during the night period in a ventral SCN neuron. *E*_GABA_ for this cell was −33 mV (*I*-*V* curve below).

Inspection of the histograms revealed asymmetric patterns of the *E*_GABA_ between dorsal and ventral SCN and between neurons recorded during the day and those recorded during the night; therefore, we reanalyzed the data by grouping the neurons according to the SCN region and time of recording and whether the equilibrium potential was above or below −50 mV (Figure 3(c)). Of 45 neurons recorded during the day in the dorsal SCN, the *E*_GABA_ was −64 ± 3 mV in 19 neurons (42.2%) and −36 ± 2 mV in 26 neurons (57.8%; Figures 3(c), 4(a), and 5(a)), whereas, of 17 neurons recorded during the night in the same SCN region, the *E*_GABA_ was −60 ± 1 mV in 12 neurons (70.6%; Figures 3(c), 4(c), and 5(b)) and −30 ± 8 mV only in 5 neurons (29.4%). Conversely, in the ventral SCN, of 14 neurons recorded during the day, 11 (78.6%) had an *E*_GABA_ of −61 ± 2 mV (Figures 3(c), 4(b), and 6(a)) and 3 (21.4%) had an *E*_GABA_ of −37 ± 9 mV, whereas, of 21 neurons recorded in the ventral SCN region during the night, 9 (42.9%) had an *E*_GABA_ of −61 ± 3 mV and 12 neurons (57.1%) had an *E*_GABA_ of −38 ± 2 mV (Figures 3(c), 4(d), and 6(b)). These results suggest a differential regulation in [Cl^−^]_i_ depending on the region of the nucleus and ZT of recording. A Kruskal-Wallis test carried out on the data that represented the majority of each group (above or below −50 mV, Figure 3(c)) demonstrated a statistically significant difference between the groups (*H* = 42.4, *P* < 0.0001). A Dunn's multiple comparison test indicated significant differences between dorsal day versus dorsal night, dorsal day versus ventral day, dorsal night versus ventral night, and ventral day versus ventral night. (*P* < 0.05; Figure 3(d)).

As we previously stated, different research groups have identified an excitatory role of the inhibitory classical neurotransmitter in the SCN and other areas of the brain during development [25]. The most likely candidates to control [Cl^−^]_i_ are the Cl^−^ cotransporters. In the SCN several lines of evidence indicate that the NKCC1 cotransporter is the most important for [Cl^−^]_i_ regulation [22, 23]. In order to test the participation of the NKCC1 cotransporter in [Cl^−^]_i_ regulation, we used the selective blocker bumetanide (10 *μ*M). Bumetanide was added to a subpopulation of the experimental groups mentioned above. In those neurons that *E*_GABA_ was hyperpolarized (below −50 mV) bumetanide administration did not have any effect on the reversal potential (data not shown). In contrast with those neurons with a depolarized *E*_GABA_ (above −50 mV), bumetanide adding shifted the values to more negative than −50 mV (*W* = 28, *P* = 0.01, Wilcoxon test). The results are summarized in Table 2. These results are in agreement with the participation of the NKCC1 cotransporter in regulating [Cl^−^]_i_, as previously proposed [22, 23].

### 3.2. Whole Cell Patch Clamp Recordings

**Figure 7:**
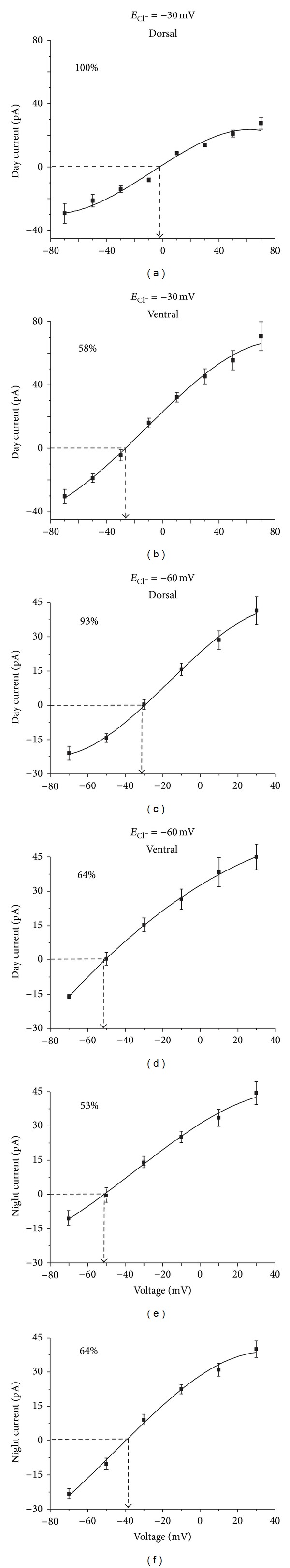
*E*_GABA_ is shifted from the *E*_Cl^−^_ estimated from the Nernst equation in subpopulations of the SCN neurons recorded in whole cell configuration. At an estimated *E*_Cl^−^_ of −30 mV, the dorsal SCN neurons recorded during the day showed an *E*_GABA_ of 0 ± 2 mV (a), while in ventral SCN the predominant *E*_GABA_ is −28 ± 3 mV (b), close to the hypothetical equilibrium potential. At an estimated *E*_Cl^−^_ of −60 mV, when recorded during day, the predominant GABAergic sPSCs equilibrium potential in the dorsal SCN was −32 ± 2.6 mV (c), while in the ventral SCN was −51 ± 1.4 mV (d). Conversely, during the night, the predominant equilibrium potential in the dorsal SCN was −53 ± 2.3 mV (e) and in the ventral SCN was −39 ± 1.9 mV (f).

In whole cell patch clamp configuration, there is dialysis of the ions contained in the patch pipette into the intracellular space, as well as wash-out of elements required for neuronal functions. However, since GABAergic sPSCs depend on *E*_Cl^−^_ we can attempt to use whole cell configuration to control (clamp) the intracellular concentration of ions and test whether SCN neurons could have a different *E*_GABA_ than the one predicted by the Nernst equation [26, 27]. To answer this question, we used two intracellular solutions with different Cl^−^ concentrations, one with an *E*_Cl^−^_ = −30 mV and another with the *E*_Cl^−^_ = −60 mV. We analyzed *E*_GABA_ in 81 SCN neurons recorded in whole cell patch clamp configuration (Figures 3(e)–3(f)). In 19 neurons recorded during the day (7 dorsal and 12 ventral) we used the internal solution with an *E*_Cl^−^_ of −30 mV. In all dorsal SCN neurons the *E*_GABA_ was 0.2 ± 2 mV, which is above the expected *E*_Cl^−^_ (Figures 3(e) and 7(a)). In contrast, in 7 of 12 neurons in the ventral SCN (58.3%) *E*_GABA_ was −28 ± 2.8 mV (close to the calculated *E*_Cl^−^_) (Figures 3(e) and 7(b)), and in the remaining 5 neurons (41.7%) the *E*_GABA_ was −4 ± 2.7 mV (nearly 30 mV above the *E*_Cl^−^_).

**Figure 8:**
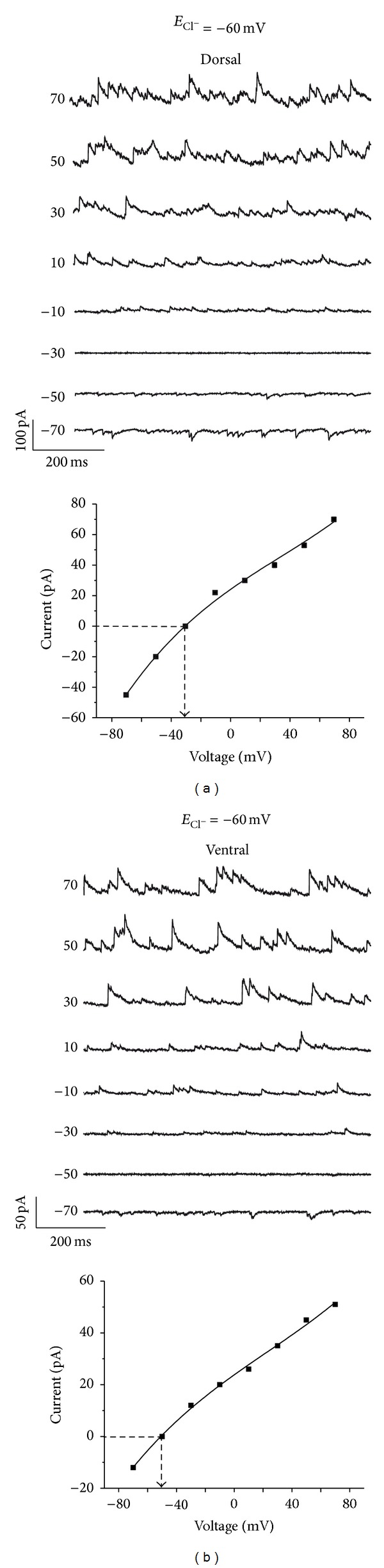
Regional differences in *E*_GABA_ obtained in whole cell configuration. (a) Dorsal SCN neuron recorded during the day in whole cell. *E*_GABA_ for this neuron was −30 mV (*I*-*V* curve below). (b) Ventral SCN neuron recorded during the day in whole cell configuration. *E*_GABA_ for this neuron was −50 mV (*I*-*V* curve below). Theoretical *E*_Cl^−^_ = −60 mV.

We used the second intracellular solution with an *E*_Cl^−^_ of −60 mV to record from 66 SCN neurons. In 14 of 15 dorsal SCN neurons recorded during midday the *E*_GABA_ was −32 ± 2.6 mV, nearly 30 mV above the *E*_Cl^−^_ (Figures 3(f), 7(c), and 8(a)); the remaining neuron had an *E*_GABA_ of −62 mV, close to the expected *E*_Cl^−^_. In contrast, 10 of 19 neurons (52.6%) recorded from the dorsal SCN during the night showed an *E*_GABA_ of −53 ± 2.3 mV, close to the *E*_Cl^−^_ (Figures 3(f) and 7(e)), whereas in the remaining 9 neurons (47.4%) the *E*_GABA_ was −36 ± 1.8 mV, about 30 mV above the *E*_Cl^−^_. In the ventral SCN, of 14 neurons recorded during midday 9 (64.3%) had an *E*_GABA_ of −51 ± 1.4 mV, close to the *E*_Cl^−^_ (Figures 3(f), 7(d), and 8(b)), whereas the remaining 5 (35.7%) had an *E*_GABA_ of −32 ± 5.7 mV, about 30 mV above the *E*_Cl^−^_. Of 14 neurons recorded in the ventral SCN during the night, only 5 (35.7%) had an *E*_GABA_ of −52 ± 0.9 mV which is close to the *E*_Cl^−^_, whereas the remaining 9 neurons (64.3%) had an *E*_GABA_ of −39 ± 2 mV (Figures 3(f) and 7(f)). The different distribution of neurons according to the *E*_GABA_ in whole cell experiments (*E*_Cl^−^_ = −60 mV; Figure 3(f)) was similar to the distribution found using perforated patch recording (Figure 3(c)). Together, these results suggest that the SCN neurons have autonomous mechanisms to increase [Cl^−^]_i_ that can be increased by the exogenous Cl^−^ concentrations contained in the recording pipette.

## 4. Discussion

In the majority of the mature neurons, GABA acts as an inhibitory neurotransmitter because [Cl^−^]_i_ is lower (~5 mM) than [Cl^−^]_o_ (~140 mM) which leads in most neurons to an *E*_Cl^−^_ hyperpolarized in comparison to the resting membrane potential (*E*_*m*_). Synaptic inhibition occurs when the activation of GABA or glycine receptors causes an inward flux of Cl^−^ ions, which in turn hyperpolarizes the neuronal *E*_*m*_. [Cl^−^]_i_ is regulated by different cotransporters: the NKCC1 cotransporter pumps Cl^−^ into the neuron, whereas the K^+^ and Cl^−^ cotransporters (KCC2 and KCC4) pump Cl^−^ out of the neuron. Thus, upregulation of NKCC1 activity or downregulation of KCC2 or KCC4 activity increases [Cl^−^]_i_ which depolarizes the *E*_Cl^−^_ with respect to the *E*_*m*_, which leads GABA to produce excitatory postsynaptic responses.

### 4.1. Perforated Patch Experiments

In this study we analyzed the *E*_GABA_ in the SCN using the perforated patch clamp technique. We found at least 2 populations of SCN neurons: 53% showed an *E*_GABA_ close to the typical *E*_Cl^−^_ in the adult brain, whereas 47% showed an *E*_GABA_ depolarized by about 30 mV as compared to the typical *E*_Cl^−^_. Since in the second set of neurons the *E*_GABA_ is depolarized with respect to −45 mV, typical *E*_*m*_ of SCN neurons [28], activation of GABA_A_ receptors (R) leads to an outward flux of chloride ions following its driving force, which depolarizes SCN neurons as previously reported [14, 15, 20–23].

We also found that the relative proportion of SCN neurons expressing each *E*_GABA_ change with time in each of the two studied regions. Thus in dorsal part of the SCN about 58% of neurons is distributed around an *E*_GABA_ of −36.2 ± 2 mV during the day and decreased to 29% during the night, while in the ventral part such a depolarized value was found only in 21% of neurons recorded during the day and increased up to 57% during the night. These changes suggest that the neuronal subsets found according to the *E*_GABA_ represent functional states from a general neuronal population. This hypothesis may conciliate seeming discrepancies among previous reports of excitatory effects of GABA in SCN neurons either during the day [14, 15, 19], or during the night [21–23].

### 4.2. Cl^−^ Cotransporter in SCN Neurons

The depolarizing shift in the *E*_GABA_ in SCN neurons is mainly due to NKCC1, as indicated by the shift back to the typical *E*_GABA_ after administration of its specific blocker bumetanide. These results corroborate previous observations on the participation of NKCC1 in the regulation of [Cl^−^]_i_ [22, 23]. However, we cannot exclude the participation of other chloride cotransporters since NKCC1, KCC2, and KCC4 are all present in SCN neurons [29].

### 4.3. Whole Cell Experiments

Although in whole cell the intracellular messengers could wash out and blunt SFR circadian rhythms [30], there is no evidence that they have major effects on transporters and ionotropic receptors in SCN neurons. Hence we further test the effect of shifting [Cl^−^]_i_ from its usual value in the SCN by the use of two different internal solutions in whole cell configuration to “clamp” *E*_Cl^−^_ to predicted values of −30 mV and −60 mV and to compare them with the actual *E*_GABA_. In these experiments we found two populations of neurons distributed around *E*_GABA_ values, one similar to the *E*_Cl^−^_ predicted from the internal solution composition and the other depolarized about 30 mV (~0 mV and ~−30 mV) from the value expected from the internal solution (−30 mV and −60 mV). These results suggest an autonomous mechanism in SCN neurons that increases [Cl^−^]_i_. Given the results obtained with bumetanide application in our perforated patch experiments and previous evidence in the field [22, 23], it is likely that the mechanisms that increase [Cl^−^]_i_ in SCN neurons are associated with the NKCC1 cotransporter. Comparable results have been found by different laboratories (using whole cell recordings), where the actual *E*_Cl^−^_ differs from the calculated one for [Cl^−^]_i_ and [Cl^−^]_o_ [26, 27]. We hypothesize that [Cl^−^]_i_ (near GABA_A_ R) was even more increased than the Cl^−^ concentration provided by the pipette recording, as a result of local mechanisms as NKCC1 cotransporter.

An interesting finding is that in the experiments with an estimated *E*_Cl^−^_ of −60 mV the SCN neurons populations showed relative distributions and dynamics regarding time and region which resemble the ones found in the perforated patch recordings. The similarity in the distribution of *E*_GABA_ in these two experimental configurations allows us to recognize a common process that is present despite the dissimilarity of the experimental conditions and the fact that the process is independently regulated in time at different SCN regions.

### 4.4. Debate about Excitatory GABA Actions

There has been debate about the role of GABA in the SCN for almost the last two decades. The majority of the studies deal with this topic in reference to the effects of agonists and antagonists of GABA_A_ R or GABA administration on the SFR of SCN neurons. The study of GABA effects on SFR allows inferring about its role on the neuronal network; however, in SCN neurons, SFR is a physiological process much more intricate than *E*_GABA_ or *E*_Cl^−^_. SFR involves activation of several types of different voltage-activated channels by phosphorylation and dephosphorylation that can mask the function of GABA. *E*_GABA_ itself might be a more accurate indicator of GABA actions during different times of the circadian cycle as well as SCN regions.

Wagner et al. [15] found excitatory actions of GABA during the day, through GABA_A_ R activation, whereas opposite result is found during the night period. Synaptic noise analysis (in whole cell experiments) indicates a higher [Cl^−^]_i_ during the day in comparison to the night. However, the authors do not describe the region of the SCN that was recorded from, which could be the reason of discrepancy between the excitation and inhibition they find in the same circadian time of SCN neurons. In this study we found excitatory actions of GABA during the day in the dorsal SCN, whereas inhibitory actions in the night period in the same area, which is consistent with the conclusions of Wagner et al. Additionally, we calculated [Cl^−^]_i_ in the perforated patch experiments using *E*_GABA_ and the Nernst equation [31]. In the dorsal SCN, during the day [Cl^−^]_i_ is ~32 mM (58%; Figure 3(c)), whereas during the night it is ~12 mM (71%; Figure 3(c)). These calculated [Cl^−^]_i_ are comparable to the [Cl^−^]_i_ that Wagner et al. found.

Liu and Reppert [12] state the role of GABA as a synchronizer in the SCN. Even though the authors report inhibitory actions of GABA at all times explored, they describe that GABA pulses induce phase shifts in the circadian rhythm of the extracellular SFR (through the activation of GABA_A_ R) of single SCN neurons, in cultured slices. The phase shifts induced by GABA are similar (Liu and Reppert, Figure 3) to the ones that result from the photic stimulation in behavioral experiments or the phase shifts that result by the administration of glutamate on the SFR of SCN neurons [32], which suggest excitatory actions of GABA on the SCN neurons. These results are consistent with our present findings regarding a depolarized *E*_GABA_ in ventral SCN neurons during the night period. As we have mentioned before, ventral SCN neurons are mainly responsible for the synchronization of external signals as light, and they transmit the phase shift information to the rest of the SCN network. We hypothesize that the putative excitatory GABA actions enforce the information communicated by light through the activation of ionotropic glutamate receptors.

In contrast to our findings, de Jeu and Pennartz [21] describe a more depolarized *E*_GABA_ (~−59 mV) during the night than during the day period (~−70 mV), in recordings obtained with perforated patch in acute SCN slices. The authors do not provide any indication about the area of the SCN where the recordings were performed. Though our results differ from these, it is interesting to notice the variability that *E*_GABA_ has during both periods of the circadian time (day and night) and the size of the samples (*N* = 17 day/*N* = 18 night). We hypothesize that with a bigger population in the results of de Jeu and Pennartz there would be more than one group in the day and night periods.

Shimura et al. [31] report a rhythmical [Cl^−^]_i_ that peaks in the middle of the day, calculated by *E*_GABA_ in perforated patch clamp experiments carried out in acutely dissociated SCN neurons. [Cl^−^]_i_ proposed by these authors (~23 mM) and the time of its peak (middle of the day) are comparable to the results found in the present work, given that we calculated a putative [Cl^−^]_i_ of ~32 mM during the day and ~12 mM during the night in dorsal SCN neurons. Shimura et al. describe also a lower [Cl^−^]_i_ (~10 mM) at other times of recording that also is in agreement with the present results.

Extracellular recordings (after several days in dissociated SCN neurons culture) show that bicuculline administration decreases the SFR during the period of higher activity (day) whereas it increases the SFR during the period of lower activity (night). These results are relevant because they indicate that the same SCN neuron can regulate GABA actions depending on the circadian time [19]. Analogous results are reported by Ikeda et al. [33] where the GABA_A_ agonist (muscimol) increases [Ca^2+^]_i_ during the day in dorsomedial SCN neurons in a higher proportion than during the night. These results are found in postnatal ages beyond P6 (P9–14), whereas at P6 and younger there are no differences between day and night. These results indicate ontogenic changes that modulate circadian GABA actions. These two studies are in accordance with the present results.

Choi et al. [22] demonstrated excitatory GABA actions mainly during the night, on the SFR that was monitored after the exogenous administration of GABA and the GABA_A_ antagonist bicuculline. However, there is evidence that relates bicuculline to the blocking of Ca^2+^-activated K^+^ channels that directly regulate the spike generation [34]. Moreover, Choi et al. found that *E*_GABA_ is more depolarized in the dorsal part of the SCN during the night period than at any other time/SCN region. These last experiments were obtained in perforated patch configuration of SCN slices. The results described by Choi et al. are in contrast with the present results. An interesting fact is that Choi et al. found excitatory GABA actions at all SCN regions explored and at all times of the circadian cycle (Figure 3(c)) [22], which is comparable to our results, though the proportion of excitatory GABA actions (regarding SCN region/time) are different from those found in the present results. We ignore how *E*_GABA_ could be integrated in the neural activity as the SFR or the excitatory postsynaptic potentials are. As we have previously stated the SFR is a more complex process than *E*_GABA_, where *E*_GABA_ is just one term in the equation. We can infer that *E*_GABA_ could have an influence on SFR, especially when spike properties of rhythmic SFR are silent or downregulated.

Irwin and Allen [23] found using Ca^2+^ imaging that GABA increases the intracellular Ca^2+^ concentration [Ca^2+^]_i_ at all circadian times and in all regions of the SCN (dorsal/ventral); however, this Ca^2+^ increase is higher in the dorsal part during the night. The increase in [Ca^2+^]_i_ that is reported in the ventral SCN during the night in comparison to the day period in the same area (Figure 4(e) Irwin and Allen [23]) is comparable with the results of this study (more depolarized *E*_GABA_ during the night in the ventral SCN) but is inconsistent with the fact that [Ca^2+^]_i_ increases even more in the night period in dorsal SCN. [Ca^2+^]_i_ could be taken as an indirect method of *E*_GABA_, given that it is expected that the Cl^−^ efflux depolarizes *E*_*m*_ and induces activation of voltage-gated Ca^2+^ channels. Excitatory actions induced by GABA have been reported by many laboratories using different approaches. Our observations have the advantage that they were obtained in acute slices (over dissociated neurons), with a direct method to estimate GABA actions (*E*_GABA_), through measurement of spontaneous GABA release rather than through administration of exogenous GABA or other GABA_A_ agonists, which may have undesirable effects on other proteins (such as GABA_B_ R, transporters, and voltage-activated channels). Moreover, our recordings were obtained with two different patch clamp configurations.

Our present results demonstrate dynamical GABA modulation which depends on the time of the day and the location of the neuron within the SCN. These results may be due to circadian modulation of NKCC or other Cl^−^ cotransporters but further studies are still necessary. Present findings suggest excitatory actions of GABA during the day in the dorsal region that might be important for the output signal of the SCN neurons to other projecting areas, especially in those neurons that have a downregulated SFR. During the night, the excitatory GABA actions are centered in the ventral SCN that could conciliate the reports that indicate phase shifts induced by GABA at a cellular level as well as behavioral level, with those indicating that light phase shifts involve NMDA receptors activation in the ventral SCN.

**Figure 9:**
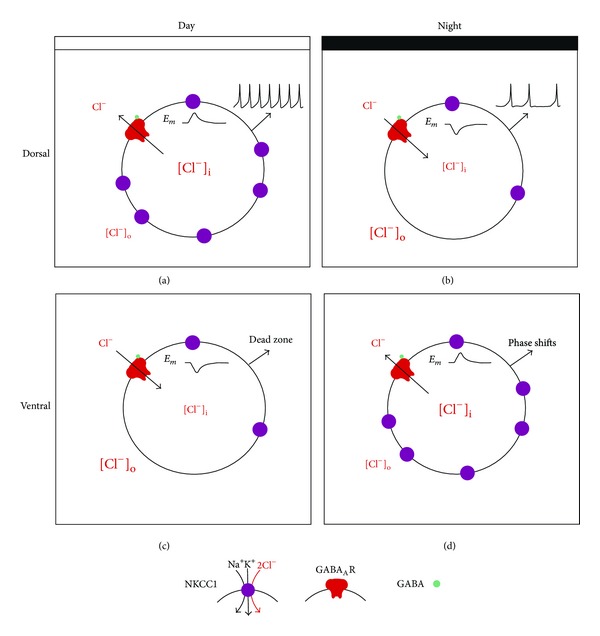
Integrative model of excitatory GABA actions depending on SCN region and circadian time. (a) Dorsal day SCN neurons. High expression of NKCC1 cotransporters increases the [Cl^−^]_i_. GABA_A_ R activation induces Cl^−^ efflux, which depolarizes the *E*_*m*_. Depolarized *E*_*m*_ increases the probability of action potentials that strengthens the SFR during the day. (b) Dorsal night. Low expression of NKCC1 decreases the [Cl^−^]_i_. GABA binding induces Cl^−^ influx, which hyperpolarizes the *E*_*m*_. More negative *E*_*m*_ decreases the probability of spikes that reduces the SFR during the night. (c) Ventral day. Diminished NKCC1 expression reduces [Cl^−^]_i_, and GABA_A_ R activation hyperpolarizes *E*_*m*_. Less excitability in ventral neurons decreases the probability of phase shifts. (d) Ventral night. Upregulated NKCC1 expression generates high [Cl^−^]_i_ that shifts the *E*_*m*_ close to the spike threshold. More excitability in ventral SCN neurons during the night enhances the probability of phase shifts by GABA.

In conclusion, we propose the model depicted in Figure 9, where, during the day, in dorsal SCN, there would be more NKCC1 transporters in the cellular membrane of the SCN neuron that increase [Cl^−^]_i_. As result of this, GABA_A_ R activation induces an efflux of Cl^−^ that depolarizes the *E*_*m*_. This process may enforce the high SFR mechanisms of SCN neurons. At the same time in ventral SCN neurons *E*_GABA_ is hyperpolarized, probably due to low expression of NKCC1. This would reinforce the lack of response to light pulses during the death zone of the phase response curve. During the night, *E*_GABA_ is depolarized in the ventral SCN due to higher expression of NKCC1. When GABA_A_ R are activated, Cl^−^ is expelled from the neuron, depolarizing the *E*_*m*_, which is important to accomplishing the synchronization process that takes place during the subjective night. During the same period in the dorsal SCN, NKCC1 expression is downregulated, which induces a low [Cl^−^]_i_. When GABA_A_ R are activated, Cl^−^ enters into the neuron, hyperpolarizing the *E*_*m*_. This situation helps with the low levels of SFR that the SCN neurons have during the night.

## Figures and Tables

**Table 1:** Descriptive statistics from GABAergic sPSCs equilibrium potentials (*E*_GABA _in mV) recorded from SCN neurons in perforated patch configuration.

Variable	Group	*n*	min	max	median
	All neurons	97	−9	−87	−51
Region	Dorsal	62	−9	−87	−49.5
	Ventral	35	−18	−81	−51
Time	Day	59	−9	−87	−50
	Night	38	−9	−81	−51

**Table 2:** SCN neurons show two *E*_GABA_ depending on the region and time of recording, as shown by the relative distribution of the equilibrium potential around −50 mV. Blockade of Cl^−^ transporter NKCCl by bumetanide affected only the potential above −50 mV (*W* = 28, *P* = 0.01, Wilcoxon test).

Region	Time	Control		+Bumetanide	
		*n*	Mean ± SEM	*n*	Mean ± SEM
Dorsal	Day	26	−35.5 ± 1.8	11	−58.5 ± 3.9
	Night	5	−30.2 ± 7.3	5	−53.4 ± 3.7

Ventral	Day	3	−36.7 ± 8.8	3	−56.7 ± 3.6
	Night	12	−37.5 ± 2.3	5	−65.1 ± 8.2

## Abbreviations

(*E*_Cl^−^_): Chloride equilibrium potential
(DNQX): Dinitroquinoxaline-2,3(1H,4H)-dione
(APV): DL-2-Amino-5-phosphonopentanoic acid
(*E*_GABA_): Equilibrium potential of GABAergic postsynaptic currents
[Cl^−^]_o_: Extracellular Cl^−^ concentration
[Ca^2+^]_i_: Intracellular Ca^2+^ concentration
[Cl^−^]_i_: Intracellular Cl^−^ concentration
(KCC): K^+^ and Cl^−^ cotransporters
(GAD): L-Glutamic acid decarboxylase
(*E*_*m*_): Membrane potential
(NKCC): Na^+^, K^+^, and Cl^−^ cotransporters
(SFR): Spontaneous firing rate
(sPSCs): Spontaneous postsynaptic currents
(SCN): Suprachiasmatic nucleus
(ZT): Zeitgeber time
(GABA): *γ*-Aminobutyric acid.
